# Child-rated versus parent-rated quality of life of community-based obese children across gender and grade

**DOI:** 10.1186/1477-7525-11-206

**Published:** 2013-12-10

**Authors:** Chia-Ting Su, Jung-Der Wang, Chung-Ying Lin

**Affiliations:** 1 Department of Occupational Therapy, College of Medicine, Fu Jen Catholic University, New Taipei, Taiwan; 2 Institute of Public Health, College of Medicine, National Cheng Kung University, National Cheng Kung University Hospital, Tainan, Taiwan; 3 Departments of Internal Medicine and Occupational and Environmental Medicine, National Cheng Kung University Hospital, Tainan, Taiwan; 4 Institute of Allied Health Sciences, College of Medicine, National Cheng Kung University, 1 University Road, Tainan 70101, Taiwan

**Keywords:** Obese, Pediatric, Quality of life, Self-report, Proxy report

## Abstract

**Background:**

Quality of life (QoL), which can be examined using self-reports or parental reports, might help healthcare providers understand obese children’s subjective well-being in several domains of life. Community-based obese children report their QoL lower than their parents do. However, the differences between child- and parent-reported QoL have neither been tested across gender and grade nor analyzed by item. This study probed the relationship between obesity and QoL item scores in children, and compared child-reported with parent-reported QoL stratified by gender and grade.

**Methods:**

One hundred eighty-seven dyads of 8- to 12-year-old children (60 obese, 127 normal-weight) and their parents were recruited. QoL was assessed using both child- and parent-reported Pediatric Quality of Life Inventory 4.0 (PedsQL) questionnaires.

**Results:**

Regression analyses showed specific difficulties with physical and emotional QoL in third- and fourth-grade obese boys (β = 0.278-0.620), and specific problems with social functioning in fifth- and sixth-grade obese girls (β = 0.337-0.411). Moreover, parents seemed unaware of the specific difficulties that their children faced (β = 0.274-0.435).

**Conclusions:**

Obese children seemed to have their difficulties from third to fifth grade, respectively, and their parents seemed unaware of them. Thus, parents need to be more aware of specific difficulties related to childhood obesity.

## Background

Obese children are likely to have physical and psychosocial difficulties [1], including poor physical fitness [2] and low self-esteem [3]. Previous studies that have reported these obesity-induced problems have designed programs to decrease the prevalence of obesity [4] and to increase children’s habitual physical activity [5,6]. In addition to these efforts from schools and society, it is important to evaluate how obese children perceive their subjective well-being in several domains of life in addition to a traditional assessment of their objective health status.

Using quality of life (QoL) to understand more about a child’s life is an increasing trend. QoL as a measure provides healthcare providers a holistic view of obese children’s subjective well-being in several domains of life [7]. This information is essential for healthcare decision-making. Recent community-based [8-10] and clinically based [11-16] studies have reported that obese children score lower than do normal-weight children on QoL assessments. Of these studies, many [8,10-17] have assessed QoL using both child self-reports (child-reported) and parent-proxy reports (parent-reported).

Examining the parent–child relationship on QoL scores is necessary to provide insights into how one assessment is related to the other and how parents’ concerns differ from their children’s [13,18]. Studies with clinical samples [11,13,14,16,17] reported a trend of inconsistency between child-reported and parent-reported QoL; that is, obese children rated their QoL better than their parents did. However, according to a recent review [18], differences exist in parent–child agreement between clinical and nonclinical samples. Moreover, our recent study [19] found that obese children from a community-based sample rated their QoL lower than their parents did, and suggested that parents of obese children from a community-based sample are less aware of their children’s difficulties.

Many studies [8-16,19] have analyzed QoL domain and total scores for insight into obese children’s subjective well-being in their daily lives. However, to the best of our knowledge, almost no studies have examined the QoL item scores to assess obese children’s subjective well-being related to physical, emotional, and social domains. Item scores provide specific difficulties that obese children face, difficulties that cannot be portrayed using the domain scores. For example, a poor physical-summary QoL score informs us that obese children have physical problems, but a low score on a specific item, such as “hard to run”, clearly indicates the specific physical problem of running. Scores based on item-derived specific difficulties rather than on domain scores, therefore, would be clearer for healthcare providers analyzing the performance of obese children. Moreover, examining parent–child agreement on each specific item also provides detailed information about parent and child disagreements about the children’s QoL. This information is essential for offering intervention programs tailored to the children’s needs and to their relationships with their parents.

Gender and age are two important factors for examining the scores of obese children and for testing the parent–child agreement of QoL scores [20-23]. Boys and girls have significantly different QoL scores: girls seem to report lower QoL scores than boys do [21]. Moreover, unlike previous studies [8-16,19] on domain scores, we suggest analyzing the relationship between weight status and each specific QoL difficulty respectively reported by obese boys and obese girls. In addition, parent–child QoL score agreement increased with age in children with a chronic illness [22,23], as did agreement between parents and their younger, healthy children [20,21]. However, these findings do not specifically focus on obese children. We therefore suggest examinations of parent–child agreement for obese children stratified by gender and age. This will allow healthcare providers to better understand how weight status correlates with QoL score differences between obese children and their parents.

The purpose of this study was to compare QoL item-scores of obese and normal-weight Taiwanese children in a community-based sample, and to analyze the relationship between child-reported and parent-reported QoL scores. Moreover, this study examined the effect of being obese on children across gender and grade in school.

## Methods

The Institutional Review Board of National Cheng Kung University Hospital approved this study.

### QoL instrument

The Pediatric Quality of Life Inventory 4.0 (PedsQL), with parallel child self-reports and parent-proxy reports, is a self-administered scale consisting of 23 items in four subscales: physical, emotional, social, and school. Each item asks the frequency of difficulties using a 5-point Likert scale, and the rating is transformed into a score of 0 (= almost always), 25 (= often), 50 (= sometimes), 75 (= almost never), or 100 (= never). Higher scores on the physical summary (physical subscale) and psychosocial summary (emotional, social, and school subscales) represent a better QoL. Detailed information on the development and use of the PedsQL, including the Chinese version, is reported elsewhere [24-28]. In addition, because item reliability and validity have been established for this instrument [29,30], the item comparisons conducted in this study seemed appropriate.

### Participants

The target participants were healthy obese and normal-weight 8- to 12-year-old children in southern Taiwan and one of their parents. Convenience sampling was used at 10 different urban and suburban schools located near National Cheng Kung University. We then sent flyers to 32 randomly selected classes at these schools. After we had sent 1052 flyers to invite participants, 520 dyads expressed interest in this study, and all signed the informed consent forms. Of the 249 dyads that completed and returned the questionnaires (response rate: 249/520 = 48%), those for 229 dyads included all values. Because our target participants were obese and normal-weight children, we finally analyzed reports from 187 of those dyads after excluding children who were classified as “overweight” (rather than “obese”) or “underweight” (rather than “normal-weight”). Weight status was based on each child’s body mass index (BMI). Table 1 shows the general BMI criteria for obese and normal-weight Taiwanese children between 8 and 12 years old [31].

**Table 1:** BMI criteria for obese and normal-weight Taiwanese children

	**BMI (kg/m**^**2**^**)**			
**Group**	**8–9 years old**	**9–10 years old**	**10–11 years old**	**11–12 years old**
Obese				
Boys	> 22.0	> 22.5	> 22.9	> 23.5
Girls	> 21.0	> 21.6	> 21.6	> 23.1
Normal-weight				
Boys	19.3–15.0	19.7–15.2	20.3–15.4	21.0–15.8
Girls	18.8–14.6	19.3–14.9	20.1–15.2	20.9–15.8

*BMI* body mass index.

### Procedures

The data were collected from October 2008 to May 2010, as previously described [19]. In brief, a trained researcher used a stadiometer and a scale to measure the children’s height and weight. The children next spent about 20 minutes completing the PedsQL child self-report in their classrooms. They then took home the PedsQL parent-proxy report and background information sheet for their parents to complete and return to the researcher within a week.

### Statistical analysis

Demographic characteristics between the two groups and between the respondents and the non-respondents were compared using independent *t* tests or χ^2^ tests. Independent *t* tests were used to compare the PedsQL child-reported and parent-reported scores between the two groups.

The discrepancies in item scores between the child-reports and parent-reports were examined using paired *t* tests. The magnitude of the discrepancies was measured using the unbiased Cohen’s effect size (*d*) as the mean difference between child-reports and parent-reports ([item scores of child self-reports] – [item scores of parent-proxy reports]) divided by the standard deviation of child-reported item scores [32]. A *d*-value > +0.2 indicated that the parents rated their children’s QoL lower than their children did, and a *d*-value < −0.2 indicated that the parents rated their children’s QoL higher than their children did [19,33].

Several regression analyses on obese children, which contained basic demographic characteristics (age, gender, and family income), were done to explore the association of basic demography and the magnitude of the score difference between the child-reports and parent-reports. We used the score differences of each subscale score and of total score as dependent variables. Age, gender, and family income were used as independent variables.

To probe the relationship between weight status and QoL, and the association between weight status and the magnitude of the child-report and parent-report difference, additional regressions with data stratified by gender and grade (viz., 3rd- and 4th-grade boys, 5th- and 6th-grade boys, 3rd- and 4th-grade girls, and 5th- and 6th-grade girls) were done. The dependent variables were the child-reported score and the difference between the child-reported and parent-reported scores on each item; the independent variables were weight status, age, and family income. In addition, an absolute value of skewness > 3 or an absolute value of kurtosis > 10 may challenge the assumption of normal distribution [34]. We used these criteria to examine the normal distribution for each item, and for each difference between child- and parent-reported scores. Finally, the absolute skewness values of all items but four (P1 = 3.167; P5 = 6.609; P6 = 3.482; So4 = 3.162), and all differences between child- and parent-reported scores were < 3. Similarly, only the same four items (P1 = 10.855; P5 = 54.649; P6 = 10.976; So4 = 12.104) and one difference between child- and parent-reported score (P5 = 22.068) did not fulfill the kurtosis criteria. All statistical analyses were done using SPSS 16.0.

## Results

The distribution of obesity and gender for our respondents (n = 187) was not significantly different from that of the non-respondents (n = 165) and the Taiwan population. However, the percentages for children in grades 5 and 6 were higher for the respondents than for the non-respondents, which makes our sample more representative for children in grades 5 and 6 (Table 2). The Obese group contained 60 dyads and the Normal-weight group contained 127 dyads. Mean BMIs were significantly (p < 0.01) different between the two groups (Table 3). In addition, the parents of obese children had significantly higher BMIs than did the parents of normal-weight children (father: p = 0.01; mother: p = 0*.*02). No other significant differences were found in participant characteristics between the two groups. None of the children had visited a physician in the past month, according to their parents’ reports. Finally, there were no significant differences in QoL between genders or between grades.

**Table 2:** Demographic comparisons between respondents and non-respondents, and between respondents and Taiwan population

	**Convenience sample**						
	**Respondents**^**b**^	**Non-respondents**^**c**^			**Taiwan**^**a**^		
	**n (%)**	**n (%)**	**χ**^**2**^	**p**	**%**	**χ**^**2**^	**p**
Gender			0.01	0.92		0.17	0.69
Boys	103 (55.1 %)	90 (54.5%)			53.6%		
Girls	84 (44.9%)	75 (45.5%)			46.4%		
Grade			38.62	<0.01		--	--
3rd	28 (15.0%)	59 (35.8%)			--		
4th	52 (27.8%)	59 (35.8%)			--		
5th	55 (29.4%)	34 (20.6%)			--		
6th	52 (27.8%)	13 (7.9%)			--		
Weight status			0.23	0.63		2.45	0.12
Obese	60 (32.1%)	49 (29.7%)			27.0%		
Normal	127 (67.0%)	116 (70.3%)			73.0%		

^a^Compared with respondents, and data were based on Chu and Pan [31].

^b^Overweight (n = 34) children were not included.

^c^Overweight (n = 41) and underweight (n = 52) were not included.

**Table 3:** Frequency distributions of demographic characteristics of participants and their parents

	**Obese**		**Normal-weight**		**p**
	***n***	**Mean (SD)**	***n***	**Mean (SD)**	
Gender					0.03*
Girl	20	--	64	--	
Boy	40	--	63	--	
Age (years)	60	10.5 (1.3)	127	10.8 (1.2)	0.17
Height (cm)	60	145.1 (9.8)	127	144.2 (9.6)	0.54
Weight (kg)	60	53.7 (9.6)	127	37.7 (7.1)	< 0.01*
BMI (kg/m^2^)	60	25.3 (2.1)	127	17.9 (1.5)	< 0.01*
Rater^a^					0.20
Mother	31	--	78	--	
Father	28	--	40	--	
Other	0	--	7	--	
Father’s age (years)	51	43.7 (5.8)	95	41.9 (7.2)	0.13
Mother’s age (years)	49	39.5 (5.7)	98	39.8 (5.5)	0.72
Father’s education					0.61
≤ K-12	32	--	62	--	
> 12 years	19	--	44	--	
Mother’s education					0.81
≤ K-12	28	--	61	--	
> 12 years	21	--	42	--	
Father’s BMI	49	26.1 (3.9)	87	24.4 (2.8)	0.01*
Mother’s BMI	46	22.5 (3.8)	92	21.2 (2.6)	0.02*
Family income (US$/month)					0.65
≤ 699	8	--	13	--	
700-1699	20	--	37	--	
≥ 1700	22	--	45	--	

*BMI* body mass index.

*p < 0.05.

^a^The raters of parent-reported PedsQL, and the cells of “father” and “other” were combined in the χ^2^ test because two cells (33.3%) had less than the expected count of 5.

### Comparing child-reported and parent-reported PedsQL scores for obese children

There were significant differences in 4 items (50%) of the physical subscale, in 2 items (40%) of the emotional subscale, and in 1 item (20%) of the social subscale. Based on Cohen’s *d*, we found that the parent-reported ratings of obese children’s QoL were higher than the child-reported ratings for 5 items (P1, P2, P3, P7, and P8), 3 items (E2, E4, and E5), and 2 items (So 3 and So5) on the physical, emotional, and social subscales, respectively. Moreover, one item (P6) on the physical subscale showed that the parent-reported rating was lower than the child-reported rating (Table 4).

**Table 4:** Comparisons between child- and parent-reported PedsQL for obese children (n = 60)

**Item#**	**Difference**^**a**^	**t**	**p**	***d***^**b**^
P1: Hard to walk more than a block	−5.00	−1.99	0.051	**−0.25**
P2: Hard to run	−12.50	−3.19	< 0.01*	**−0.46**
P3: Hard to do sports or exercise	−9.17	−2.57	0.01*	**−0.35**
P4: Hard to lift something heavy	0.84	0.25	0.80	0.04
P5: Hard to take bath or shower	−0.42	−0.19	0.85	−0.03
P6: Hard to do chores around house	11.25	3.14	< 0.01*	0.79
P7: hurt or ache	−5.42	−1.54	0.13	−**0.22**
P8: Low energy	−8.75	−2.31	0.03*	−**0.27**
E1: Fell afraid or scared	−5.42	−1.37	0.18	−0.18
E2: Feel sad or blue	−7.50	−1.94	0.06	−**0.25**
E3: Feel angry	−5.00	−1.33	0.19	−0.17
E4: Trouble sleeping	−10.00	−2.56	0.01*	−**0.32**
E5: Worry about what will happen	−11.67	−2.74	< 0.01*	−**0.37**
So1: Trouble getting along with peers	−5.08	−1.41	0.17	−0.18
So2: Other kids not wanting to be friend	−4.66	−1.07	0.29	−0.16
So3: Teased	−5.83	−1.55	0.13	−**0.21**
So4: Not doing things other peers do	1.25	0.40	0.69	0.07
So5: Hard to keep up when play with others	−7.08	−2.43	0.02*	−**0.31**
Sc1: Hard to concentrate	4.17	1.11	0.27	0.15
Sc2: Forget things	5.00	1.17	0.25	0.18
Sc3: Trouble keeping up with school work	2.09	0.61	0.55	0.10
Sc4: Miss school, not well	0.00	0.00	1.00	0.00
Sc5: Miss school, doctor appointment	0.41	0.15	0.88	0.02

*p < 0.05; In bold, *d* < −0.2; Underlined, *d* > 0.2.

^a^Difference = [child-reported PedsQL score] − [parent-reported PedsQL score].

^b^Cohen’s *d* = ([child-reported PedsQL score] − [parent-reported PedsQL score])/[child-reported PedsQL SD].

### Basic demography for the child-reported and the parent-reported differences in PedsQL scores for obese children

Age, gender, and family monthly income were used to explain the child-reported and the parent-reported differences for obese children. Only family income significantly explained the differences in the PedsQL total score (β = −0.393, p < 0.01) and on the physical (β = −0.327, p = 0.03), emotional (β = −0.342, p = 0.02), and social (β = −0.303, p = 0.04) subscales. Our results indicated that parents with higher incomes were more likely to overestimate their obese children’s QoL.

### The difficulties that obese children face

After controlling for potential confounders (viz., age and family income), regression analyses showed that obese boys in grades 3 and 4 had more difficulties running, playing sports, and doing exercise, and were less energetic than their normal-weight counterparts. Moreover, they more easily felt sad, had more trouble sleeping, and had more difficulty making friends than did normal-weight boys. The parents of the obese boys overestimated their children’s ability to run and were not aware of their children’s sad feelings. In contrast, obese boys in grades 5 and 6 did not show these difficulties (Table 5).

**Table 5:** **The impact of weight status**^**a **^**on child-reported item scores and on child-proxy differences item scores**

	**Child-reported score**				**Score difference between children and parents**			
	**Weight status β (Increased R**^**2**^**)**				**Weight status β (Increased R**^**2**^**)**			
**Dependent variable: item#**^**b**^	**Grades 3 and 4**		**Grades 5 and 6**		**Grades 3 and 4**		**Grades 5 and 6**	
Boys	n = 52		n = 51		n = 52		n = 51	
P2: Hard to run	−0.620***	(0.383)	−0.304	(0.085)	−0.435**	(0.188)	−0.112	(0.012)
P3: Hard to do sports or exercises	−0.408**	(0.166)	−0.158	(0.023)	−0.233	(0.054)	−0.013	(0.000)
P8: Low energy	−0.373**	(0.139)	−0.088	(0.007)	−0.213	(0.045)	−0.032	(0.001)
E2: Feel sad or blue	−0.343*	(0.117)	−0.199	(0.037)	−0.294*	(0.086)	−0.056	(0.003)
E4: Trouble sleeping	−0.278*	(0.077)	0.295	(0.080)	−0.274*	(0.075)	0.242	(0.054)
So2: Other kids don’t want to be friends	−0.379**	(0.143)	−0.029	(0.001)	−0.303*	(0.091)	0.017	(0.000)
So3: Teased	−0.272	(0.074)	0.075	(0.005)	−0.300*	(0.090)	0.008	(0.000)
So5: Hard to keep up when playing with peers	−0.263	(0.069)	−0.061	(0.003)	−0.127	(0.016)	−0.050	(0.002)
Girls	n = 28		n = 56		n = 28		n = 56	
P2: Hard to run	−0.290	(0.077)	−0.089	(0.008)	−0.377	(0.129)	−0.141	(0.019)
P3: Hard to do sports or exercises	−0.159	(0.023)	−0.186	(0.034)	−0.087	(0.007)	−0.234	(0.054)
P8: Low energy	−0.065	(0.004)	−0.160	(0.025)	−0.013	(0.000)	−0.191	(0.036)
E2: Feel sad or blue	−0.168	(0.026)	0.012	(0.000)	−0.189	(0.032)	0.125	(0.015)
E4: Trouble sleeping	0.053	(0.003)	−0.099	(0.010)	−0.173	(0.072)	−0.129	(0.016)
So2: Other kids don’t want to be friends	0.075	(0.005)	−0.337*	(0.112)	−0.025	(0.001)	0.137	(0.018)
So3: Teased	0.151	(0.021)	−0.411**	(0.167)	0.139	(0.018)	0.028	(0.001)
So5: Hard to keep up when playing with peers	−0.122	(0.013)	−0.350*	(0.121)	−0.186	(0.032)	−0.323*	(0.102)

*p < 0.05; **p < 0.01; ***p < 0.001.

^a^Reference group was Normal-weight, and two confounding variables (age and family income) were controlled.

^b^Items with no significant predictor were omitted.

Obese girls in grades 5 and 6 had more difficulties making friends and keeping up with peers when playing, and they were teased more often than were normal-weight girls. Parents of 5th- and 6th-grade obese girls were unaware of their children’s difficulties while playing. In contrast, 3rd- and 4th-grade obese girls did not show these problems (Table 5).

## Discussion

To the best of our knowledge, this is the first study on the differences between child-reported and the parent-reported PedsQL item scores for obese children from a community-based sample. Moreover, regression analyses stratified by gender and grade were examined for a more complete understanding of the relationship between QoL and being obese. One study [19] indicates that, based on the PedsQL domain score, the parents of obese children overrated their children’s physical ability, were unaware of their children’s emotions, and were unaware of the extent to which these results are reflected in specific behaviors (e.g., hard to run; feel sad or blue). Our data extend these findings to specific difficulties that obese boys and obese girls respectively faced and how their parents perceived these difficulties.

Our finding that the parents of obese children tended to overestimate their children’s QoL contradicts the results of published clinical studies [11-16], but agrees with published community-based studies [10,19]. One possible reason is that the parents of obese children from a community-based sample have different perspectives from the parents of obese children from a clinically based sample. Parents of children with illnesses tend to rate their children’s QoL lower than their children do, because the parents recognize and possibly overestimate the effects of the illnesses once they are brought to medical attention and verified by physicians [35]. Thus, the parents of obese children from a community-based sample may overestimate their children’s QoL because they are unaware of their children’s obesity-induced problems.

To probe this issue, we examined the differences in item scores between child-reported and parent-reported PedsQL. Our findings indicated that parents were not aware of their obese children’s low level of athletic ability, such as running and playing sports [2,36], their physical pain, their emotional problems [3], their trouble sleeping, and their social difficulties (viz., being teased and having difficulty physically keeping up with their peers [36]). However, parents gave their obese children a lower score on item P6 (Hard to do chores) than their children did. This finding agrees with our previous study [28] on healthy children, and suggests that parents may rate item P6 lower than their children do because parents evaluate their children’s ability to follow orders to do housework rather than their ability to actually do the chores.

Moreover, we examined the effect of basic demography. A surprising result indicated that family income was a potential explanatory factor for these differences: the QoLs of obese children whose parents earned a higher income were likely to be overrated than those of obese children whose parents earned a lower income. This finding suggests that healthcare providers should consider family income when addressing child–parent agreement on QoL. However, our results cannot explain the relationship between family income and child-reported and parent-reported differences. Additional studies on this issue are needed to understand what factors might mediate the relationship between family income and child-reported and parent-reported differences.

Furthermore, after potential confounders had been controlled for, we found that 3rd- and 4th-grade obese boys had several physical and emotional difficulties, and that 5th- and 6th-grade obese girls had some social difficulties. These findings are in accord with Steinsbekk et al. [37], who reported that the elevated levels of psychopathology in obese children contributed to their impaired parent-reported QoL. Traditionally, boys are expected to be muscular, independent, and tough [38-40]. However, because obese boys are substantially overweight, they generally do not have strong bodies (“are not muscular”) or minds (“are not independent and tough”) [2,3], which probably affects how they rate their physical and emotional functioning. Likewise, girls need friends to share their emotions and to play group-oriented roles [38,40]. However, obese girls are generally not welcomed by their peers, and are teased about their weight during puberty [3,41,42]. Thus, obese girls may feel frustrated by social interaction. This information is helpful for healthcare providers who plan intervention programs tailored to the children’s needs. For example, physical training and emotional support are important for obese 3rd- and 4th-grade boys, and social interaction groups for obese 5th- and 6th-grade girls.

Of these specific difficulties, we found that parents overestimated their obese boys’ athletic ability, such as running, and were unaware of their sad moods and sleeping problems. In addition, parents were unaware of their obese girls’ relationships with peers. Because most parents in Taiwan work full-time, they have little time to spend with their children [43]; therefore, they may not detect some warning signs from their children. The difficulties that community-based obese children face are usually not as serious as those faced by clinically based obese children, but they may merely be the beginning of their problems because of their obesity. Prepubescent and pubescent children between 8 and 12 years old are at a critical stage of development; therefore, parents’ understanding, support, and guidance are crucial for establishing a stronger basis for future development and fostering a better QoL. Hence, we suggest that the parents of obese children, especially of 3rd- and 4th-grade obese boys and of 5th- and 6th-grade obese girls, become more aware of their children’s difficulties and provide support to help them deal with these difficulties.

Shaya et al. [4] report that many studies on obese children have examined the intervention effects of losing or controlling weight. However, in addition to these objectively measured health indices, child-reported and parent-reported assessments of QoL are also important indicators of children’s well-being. We therefore suggest another direction for healthcare providers: address the child–parent incongruence. It is essential for healthcare providers to help parents of obese children understand the QoL problems that their children face. We suggest that parents of 3rd- and 4th-grade obese boys focus on their children’s physical and emotional performances, while parents of 5th- and 6th-grade obese girls focus on their children’s social performance. We expect that if parents understand their children’s difficulties, they will be more likely to care about and deal with their children’s obesity-related problems.

Another implication of our findings is related to using parent-proxy questionnaires. We found that parent-reported QoL differed from child-reported QoL. Therefore, we suggest that healthcare providers need to be careful when interpreting parent-reported QoL, especially for those from community-based samples.

The current study analyzed the same data our previous study [19] did. Therefore, we summarize the differences between them as follows: (1) our previous study focused on QoL domain scores. In contrast, the current study analyzed QoL item scores, which provide health professionals more specific and concrete information about QoL; (2) the current study analyzed the relationship between weight status and each specific QoL difficulty that obese children had, which is not reported in the previous study; (3) the current study separately analyzed QoL across gender and grade in school. Thus, it provides healthcare professionals clear guidelines for therapeutic intervention. Had we merely pooled all the participants together for an analysis, as was done in the previous study, we would know only that obese children have a lower QoL than do normal-weight children. Therefore, our findings in this current study seem to be more clinically useful; (4) the current study found that family income was correlated with the QoL rating difference between obese children and their parents, which the previous study did not examine.

## Limitations

This study has several limitations. First, our sample size of 187 dyads was not large enough to represent a community sample, and may, therefore, have a sampling bias. Because our sample was from southern Taiwan and near National Cheng Kung University, our results may not be representative for all of Taiwan. In addition, with a low response rate of 48%, our participants were the children whose parents signed the informed consents and completed the PedsQL, which implied that these parents care a great deal about their children. Therefore, the participants in our study may have a higher QoL than other obese Taiwanese children [44]. As a result, the representativeness of our study would be limited. However, we compared the obesity rate of the children in this study (boys: 32.5%; girls: 20.4%) with that of the Taiwan population (boys: 30.5%; girls: 23.5%) [31], and found no significant differences (boys: χ^2^ = 0.314, p = 0.575; girls: χ^2^ = 0.521, p = 0.470). In addition, Table 2 also shows that, in our sample, the rate of obesity between the respondents and non-respondents was similar. Therefore, we believe that the limited representativeness of our sample may be somewhat compensated based on our clear case–control design. Second, because the study recruited only 8- to 12-year-old children; the results may not be applicable to children younger than 8 or older than 12. Third, a cross-sectional design was used in this study, and the chronic effects of obesity on children’s QoL were not determined. The effects of being obese on children’s mental well-being, QoL, and social life, and on their relationships with their parents need longitudinal studies to clarify. Fourth, although our sample size (n = 28–56) is acceptable for regression analysis [45], some dependent variables were not normally distributed, which may influence our results. Thus, additional studies with larger sample sizes are recommended.

## Conclusions

We found that community-based obese children in Taiwan rated their QoL lower than did normal-weight children, but that their parents did not. Moreover, the parents of the obese children were unaware of several physical, emotional, and social functioning difficulties that their children had. For example, we found that obese 3rd- and 4th-grade boys faced more physical and emotional difficulties than did 5th- and 6th-grade boys. Based on our findings, healthcare providers may want to plan physical training programs and social interaction groups for obese 3rd- and 4th-grade boys and obese 5th- and 6th-grade girls, respectively. Moreover, our results suggest that when using parent-proxy assessments alone to determine the QoL of obese children, some of their difficulties may not be detected. Therefore, more effort is needed to increase parental awareness of the problems that their obese children have.

## Abbreviations

BMI: Body mass index; PedsQL: The pediatric quality of life inventory 4.0; QoL: Quality of life.

## Competing interests

The authors declare that they have no competing interests.

## Authors’ contributions

C-TS designed the study, interpreted the data, and revised the manuscript. J-DW revised the manuscript. C-YL acquired and analyzed the data, and drafted the manuscript. All authors revised and approved the final manuscript.

## References

[B1] Tsiros MD Olds T Buckley JD Grimshaw P Brennan L Walkley J Hills AP Howe PR Coates AM Health-related quality of life in obese children and adolescents Int J Obes 2009 11 387 400 10.1038/ijo.2009.42 19255583

[B2] Chen W Lin CC Peng CT Li CI Wu HC Chiang J Wu JY Huang PC Approaching healthy body mass index norms for children and adolescents from health-related physical fitness Obes Rev 2002 11 225 232 10.1046/j.1467-789X.2002.00064.x 12164476

[B3] Danielsen YS Stormark KM Nordhus IH Mæhle M Sand L Ekornås B Pallesen S Factors associated with Low self-esteem in children with overweight Obes Facts 2012 11 722 733 10.1159/000338333 23108439

[B4] Shaya FT Flores D Gbarayor CM Wang J School-based obesity interventions: a literature review J Sch Health 2008 11 189 196 10.1111/j.1746-1561.2008.00285.x 18336677

[B5] Hartmann T Zahner L Puhse U Puder JJ Kriemler S Effects of a school-based physical activity program on physical and psychosocial quality of life in elementary school children: a cluster-randomized trial Pediatr Exerc Sci 2010 11 511 522 21242601 10.1123/pes.22.4.511

[B6] Oliver M Schofield G McEvoy E An integrated curriculum approach to increasing habitual physical activity in children: a feasibility study J Sch Health 2006 11 74 79 10.1111/j.1746-1561.2006.00071.x 16466470

[B7] Lin C-Y Su C-T Ma H-I Objectively measured physical activity patterns and quality of life of overweight boys: a preliminary study Hong Kong J Occup Ther 2012 11 31 37 10.1016/j.hkjot.2012.06.001

[B8] Friedlander SL Larkin EK Rosen CL Palermo TM Redline S Decreased quality of life associated with obesity in school-aged children Arch Pediatr Adolesc Med 2003 11 1206 1211 10.1001/archpedi.157.12.1206 14662577

[B9] Swallen KC Reither EN Haas SA Meier AM Overweight, obesity and health-related quality of life among adolescents: the national longitudinal study of adolescent health Pediatrics 2005 11 340 347 10.1542/peds.2004-0678 15687442

[B10] Williams J Wake M Hesketh K Maher E Waters E Health-related quality of life of overweight and obese children JAMA 2005 11 70 76 10.1001/jama.293.1.70 15632338

[B11] Hughes AR Farewell K Harris D Reilly JJ Quality of life in a clinical sample of obese children Int J Obes 2007 11 39 44 10.1038/sj.ijo.0803410 16733522

[B12] Schwimmer JB Burwinkle TM Varni JW Health-related quality of life of severely obese children and adolescents JAMA 2003 11 1813 1819 10.1001/jama.289.14.1813 12684360

[B13] Zeller MH Modi AC Predictors of health-related quality of life in obese youth Obesity 2006 11 122 130 10.1038/oby.2006.15 16493130

[B14] Pinhas-Hamiel O Singer S Pilpel N Fradkin A Modan D Reichman B Health-related quality of life among children and adolescents: associations with obesity Int J Obes 2006 11 267 272 10.1038/sj.ijo.0803107 16231035

[B15] Janicke DM Marciel KK Ingerski LM Novoa W Lowry KW Sallinen BJ Silverstein JH Impact of psychosocial factors on quality of life in overweight youth Obesity 2007 11 1799 1807 10.1038/oby.2007.214 17636099

[B16] Ingerski LM Janicke DM Silverstein JH Brief report: quality of life in overweight youth - the role of multiple informants and perceived social support J Pediatr Psychol 2007 11 869 874 10.1093/jpepsy/jsm026 17488775

[B17] Ul-Haq Z Mackay DF Fenwick E Pell JP Meta-analysis of the association between body mass index and health-related quality of life among children and adolescents, assessed using the pediatric quality of life inventory index J Pediatr 2013 11 280 286 10.1016/j.jpeds.2012.07.049 22959137

[B18] Upton P Lawford J Eiser C Parent–child agreement across child health-related quality of life instruments: a review of the literature Qual Life Res 2008 11 895 913 10.1007/s11136-008-9350-5 18521721

[B19] Lin C-Y Su C-T Wang J-D Ma H-I Self-rated and parent-rated quality of life for community-based obese and overweight children Acta Paediatr 2013 11 e114 e119 10.1111/apa.12108 23237405

[B20] Cremeens J Eiser C Blades M Factors influencing agreement between child self-report and parent proxy-reports on the Pediatric Quality of Life Inventory 4.0 (PedsQL) generic core scales Health Qual Life Outcomes 2006 11 58 10.1186/1477-7525-4-58 16942613 PMC1564004

[B21] Jozefiak T Larsson B Wichstrom L Mattejat F Ravens-Sieberer U Quality of life as reported by school children and their parents: a cross-sectional survey Health Qual Life Outcomes 2008 11 34 10.1186/1477-7525-6-34 18489777 PMC2409303

[B22] Annett RD Bender BG DuHamel TR Lapidus J Factors influencing parent reports on quality of life for children with asthma J Asthma 2003 11 577 587 10.1081/JAS-120019030 14529108

[B23] Ronen GM Streiner DL Rosenbaum P Canadian pediatric epilepsy network: health-related quality of life in children with epilepsy: development and validation of self-report and parent proxy measures Epilepsia 2003 11 598 612 10.1046/j.1528-1157.2003.46302.x 12681011

[B24] Varni JW Burwinkle TM Seid M Skarr D The PedsQL 4.0 as a pediatric population health measure: feasibility, reliability, and validity Ambul Pediatr 2003 11 329 341 10.1367/1539-4409(2003)003<0329:TPAAPP>2.0.CO;2 14616041

[B25] Varni JW Seid M Knight TS Uzark K Szer IS The PedsQL 4.0 Generic Core Scales: sensitivity, responsiveness, and impact on clinical decision-making J Behav Med 2002 11 175 193 10.1023/A:1014836921812 11977437

[B26] Varni JW Seid M Kurtin PS PedsQL 4.0: reliability and validity of the pediatric quality of life inventory version 4.0 Generic core scales in healthy and patient populations Med Care 2001 11 800 812 10.1097/00005650-200108000-00006 11468499

[B27] Lin C-Y Luh W-M Yang A-L Su C-T Wang J-D Ma H-I Psychometric properties and gender invariance of the Chinese version of the self-report pediatric quality of life inventory version 4.0: short form is acceptable Qual Life Res 2012 11 177 182 10.1007/s11136-011-9928-1 21574017

[B28] Lin C-Y Luh W-M Cheng C-P Yang A-L Su C-T Ma H-I Measurement equivalence across child self-reports and parent-proxy reports in the Chinese version of the pediatric quality of life inventory version 4.0 Child Psychiatry Hum Dev 2013 11 583 590 10.1007/s10578-012-0352-8 23242709

[B29] Jafari P Bagheri Z Ayatollahi SMT Soltani Z Using Rasch rating scale model to reassess the psychometric properties of the Persian version of the PedsQL 4.0 Generic Core Scales in school children Health Qual Life Outcomes 2012 11 Jafari P, Bagheri Z, Ayatollahi SMT, Soltani Z 27 22414135 10.1186/1477-7525-10-27 PMC3353856

[B30] Kook SH Varni JW Validation of the Korean version of the pediatric quality of life inventory 4.0 generic core scales in school children and adolescents using the Rasch model Health Qual Life Outcomes 2008 11 41 10.1186/1477-7525-6-41 18518951 PMC2459148

[B31] Chu N-F Pan W-H Prevalence of obesity and its comorbidities among schoolchildren in Taiwan Asia Pac J Clin Nutr 2007 11 Suppl 2 601 607 17724001

[B32] Portney LG Watkins MP Foundations of Clinical Research: Applications to Practice 2000 Upper Saddle River, NJ: Prentice Hall Health

[B33] Robitail S Simeoni MC Ravens-Sieberer U Bruil J Auquier P Children proxies’ quality-of-life agreement depended on the country using the European KIDSCREEN-52 questionnaire J Clin Epidemiol 2007 11 469 478 17419958 10.1016/j.jclinepi.2006.09.007

[B34] Kline RB Principles and Practice of Structural Equation Modeling 1998 New York: Guilford Press

[B35] Eiser C Morse R Can parents rate their child’s health-related quality of life? results of a systematic review Qual Life Res 2001 11 347 357 10.1023/A:1012253723272 11763247

[B36] Puhl RM Latner JD Stigma, obesity, and the health of the nation’s children Psychol Bull 2007 11 557 580 17592956 10.1037/0033-2909.133.4.557

[B37] Steinsbekk S Jozefiak T Ødegård R Wichstrøm L Impaired parent-reported quality of life in treatment-seeking children with obesity is mediated by high levels of psychopathology Qual Life Res 2009 11 1159 1167 10.1007/s11136-009-9535-6 20131477

[B38] Adler PA Kless S Adler P Socialization to gender roles: popularity among elementary school boys and girls Sociol Educ 1992 11 169 188 10.2307/2112807

[B39] Ricciardelli LA McCabe MP Cash TF, Smolak L Body image development in adolescent boys Body Image: A Handbook of Science, Practice, and Prevention 2011 New York: Guilford Press 85 92

[B40] Davis SN Sex stereotypes in commercials targeted toward children: a content analysis Sociol Spectrum 2003 11 407 424 10.1080/02732170309220

[B41] Neumark-Sztainer D Cash TF, Smolak L Obesity and body image in youth Body Image: A Handbook of Science, Practice, and Prevention 2011 New York: Guilford Press 180 188

[B42] Wertheim EH Paxton SJ Cash TF, Smolak L Body image development in adolescent girls Body Image: A Handbook of Science, Practice, and Prevention 2011 New York: Guilford Press 76 84

[B43] Child Welfare League Foundation Love children 333: a survey of children’s perceived family warmth. Article in Chinese Child Welfare League Found Bimonthly J 2011 11 1 8 Available at http://www.children.org.tw/upload/doc/138.pdf. Accessed 20 June 2013

[B44] Lin C-Y Luh W-M Cheng C-P Yang A-L Ma H-I Evaluating the wording effect and psychometric properties of the kid-KINDL: using the multitrait-multimethod approach Eur J Psychol Assess In press

[B45] Howell DC Multiple regression Statistical Methods for Psychology 2007 Belmont, CA: Thomson Wadsworth 506

